# The Intervention Effect of SMS Delivery on Chinese Adolescent’s Physical Activity

**DOI:** 10.3390/ijerph16050787

**Published:** 2019-03-04

**Authors:** Patrick W. C. Lau, Amanda J. Pitkethly, Beeto W. C. Leung, Erica Y. Lau, Jing-Jing Wang

**Affiliations:** 1 Department of Sport & Physical Education, Hong Kong Baptist University, Kowloon Tong, Hong Kong, China; wclau@hkbu.edu.hk; 2 Sport, Exercise and Health Sciences, Edinburgh Napier University, Scotland EH11 4BN, UK; a.pitkethly@napier.ac.uk; 3 School of Arts and Humanities, Tung Wah College, Hong Kong, China; beetoleung@twc.edu.hk; 4 Department of Family Practice, University of British Columbia, Vancouver, BC V5Z 1M9, Canada; lau.y.erica@gmail.com; 5 Mass Sports Research Center, China Institute of Sport Science, Beijing 100061, China

**Keywords:** mHealth, children’s physical activity, text message

## Abstract

To examine the effects of short messaging service (SMS) frequency and timing on the efficacy of an SMS-intervention for Hong Kong Chinese adolescents, sixty nine students aged between 12 and 16 (mean age 13.75 ± 0.90) were recruited from five schools in Hong Kong. Participants were randomly assigned into one of five groups: high-frequency + self-selected timing (HST), low-frequency + self-selected timing (LST), high-frequency + assigned timing (HAT), low-frequency + assigned timing (LAT) and the control group. The total duration of the intervention was four weeks. No significant intervention effects were detected in adolescent’s PA among the five groups (F = 1.14, *p* = 0.346). No significant differences were observed in the stage movement among the five groups (*χ*^2^ = 6.18, *p* = 0.627). No significant differences appeared in the exercise benefits, barriers and benefits/barriers differential scores. However, a growth trend in the exercise benefits score in the LST and HAT groups was found in contrast to the downswing in the control group. The exercise barriers score in the HST group showed the largest reduction after intervention. The benefits/barriers differential score in all the intervention groups increased, whereas it decreased in the control group. Although an increase is demonstrated in the high dosage SMS frequency and timing, no significant intervention effects were found among the five groups in PA behavior, stage of change and exercise benefits and barriers among Hong Kong Chinese adolescents.

## 1. Introduction

### 1.1. Physical Activity and Obesity

Regular physical activity (PA) is associated with a reduced risk of obesity and type II diabetes mellitus in adolescents, as well as a reduced risk of diseases that develop later in life, such as breast cancer, hypertension, coronary heart disease and osteoporosis [1,2]. However, despite the well-established and documented benefits of PA, over half of the young population around the world does not engage in sufficient PA to achieve the potential significant health benefits [3]. The Boys’ and Girls’ Clubs Association of Hong Kong (BGCA) (2018) [4], has revealed that 59.1% of 6 to 17-year-olds in Hong Kong engaged in less than six hours of moderate to vigorous intensity activity weekly. The recommended level for this age group is 60 min or more of moderate-to-vigorous intensity PA on most days of the week. Another recent questionnaire survey revealed that less than 10% of 7 to 19-year-old self-reported participation in at least 60 min of moderate-to-vigorous physical activity (MVPA) a day. [5]. Therefore, innovative approaches to promote PA to adolescents are of paramount importance.

### 1.2. PA Intervention with Short Message Service (SMS)

Over the last decade, numerous physical activity (PA) interventions have been developed and implemented. However, many are ineffective [6], and traditional PA interventions, which are typically delivered face-to-face, may not be affordable for large-scale use. However, information communication technology (ICT), more specifically, Short Message Services (SMS) via mobile phones, is considered a promising intervention delivery method by which to influence and potentially increase behavioral change [7,8]. SMS is a text messaging service component of most telephone, internet, and mobile-device systems. It is a most widely used data application. The intervention effect of SMS on different health behaviors including physical and mental health has been supported in previous studies in which age, ethnicity and socio-economic status are considered [9,10]. Additionally, SMS is also fast, portable, convenient and inexpensive, and has a wider reach than other forms of communication [9]. Because the mHealth technologies are currently making considerable advances in PA behavioral change, SMS could play a more important role within PA behavioral change interventions if the functions are further explored and maximized.

The reminder effect through SMS to the participants is the most obvious function in achieving a significant intervention effect in previous studies [11,12]. Conversely, SMS symbols could develop a closer relation, better outreach, and in-group culture when communicating with peers. Delivery of SMS could also reduce the barriers (i.e., data tracking and analysis) associated with treatments or services provided to the patients in medical settings [13]. SMS produced a more motivational, interactive and social support effect on the participants [14,15,16]. SMS is also an inexpensive and automated communication technology in which the message can be “pushed” to the receiver, and he/she would not consider rejecting it. This automated communication reaction enhances the probability of success of the intervention.

Currently, among adolescent mobile phone users, SMS is an integral part of daily activities, enabling the maintenance of social networks and the expression of feelings [17,18,19]. More importantly, from an adolescent perspective, SMS is considered among young people as a fashionable means of reflecting individual identity and culture within peer groups, for example, through the increased use of personalized languages and emotional symbols, i.e., emoji [18,19].

The use of mobile phone technology has been widely adopted in chronic disease management programs such as diabetes [20], asthma [21,22], sexual health [23], smoking cessation [24,25], alcoholism, drug abuse and weight loss [26] and has consistently demonstrated promising results in acting as an adjunct to existing treatment, increasing awareness, and improving compliance with medication and adherence to treatment programs.

The use of mobile technology for improved PA levels has primarily focused on adult populations [27,28]. However, recently an increasing number of studies have implemented mobile to promote PA behaviors among adolescents [29,30]. A review by Lau et al. [31] found support for the use of SMS as a medium to improve PA among children and adolescents; however, six of the nine studies included in the review demonstrated satisfactory methodological quality.

### 1.3. The Theoretical Framework of the Study

The trans-theoretical model (TTM) has been one of the major models for the stage of change in past decades when investigating behavioral change among children and adults in the literature [32,33]. This stage-matched concept demonstrated significant insights regarding both PA adoption and maintenance when designing interventions for adolescents in an intervention study [33,34]. A major value of the stage of change model is that it not only demonstrates the readiness of an individual’s intention to change his or her PA behavior but also provides stage-based and stage-matched guidelines to the PA researchers [35]. Because the TTM has demonstrated effects on the improvement of PA in the student population, it would be interesting to observe whether SMS may be able to produce a significant impact on the adolescents in this study, particularly on the transition, if any, between different stages of change in PA behavior [32].

### 1.4. Research Questions of the Study

Perceived exercise benefits and barriers have been documented as significant mediators in the previous PA behavioral change studies [36,37]. Significant relations were demonstrated between PA benefits, PA barriers and the current PA habits of the participants [38]. A better understanding of the perceived benefits and barriers to PA could enhance the intervention effectiveness and efficiency [37]. The Exercise Benefits and Barriers Scale (EBBS) can help assess the changes in participant’s perceived benefits, barriers and any discrepancies. These scores would provide a better picture to understand and interpret the intervention findings in this study. It is also important to know the dynamic relations or priority between the benefits and barriers in PA when addressing adolescents’ PA behavioral change because these changes may be different when compared with different populations [39].

Because SMS-based interventions are increasing, it is essential to establish practical guidelines, particularly with respect to content and frequency. Reviews of behavior change interventions delivered by SMS [9,40] indicated that successful SMS-based health behavior change interventions were delivered at high frequency (at least 5 SMS per week) with an individually tailored SMS timing. However, these studies have all been conducted in European and American populations, and no such investigations have been conducted in Chinese population or, more specifically, in Chinese adolescent population.

In Hong Kong, a preliminary investigation supported the efficacy of promoting PA through SMS and Internet-based programs [41]. However, the effect of tailored timing and frequency has not yet been empirically examined. Existing behavior change theories have emphasized the “why” but relatively less the “how” (i.e., procedures), and behavior change interventions have not met expectations in terms of effectiveness [6]. This study’s objective was to address this issue by determining how best to integrate SMS into adolescent PA behavior. These findings may offer practical guidelines to inform the design of future SMS-based intervention for Chinese adolescent’s PA behavior. The specific purpose of this study was to examine the effects of SMS frequency and timing on the efficacy of a SMS-based intervention for Hong Kong Chinese adolescent’s PA.

The research questions were:

(1) What is the effect of the frequency and timing of the delivery of SMS impact on the quantity of adolescent’s PA?
(2) Would the stages of motivational readiness for PA be affected as a result of the SMS intervention?
(3) Would adolescents perceive a difference in benefits and barriers to PA as a result of the SMS intervention?

## 2. Materials and Methods

### 2.1. Participants

Participants were randomly recruited from five of 469 government-subsidized schools in Hong Kong, China. Sixty nine students aged between 12 and 16 (33 girls, 36 boys, age 13.75 ± 0.90) were recruited by physical education (PE) teachers from the five schools in Hong Kong. Ethical approval was granted by the Senate Committee on the Use of Human and Animal Subjects in Teaching and Research, Hong Kong Baptist University (project identification code: FRG2/10-11/046). Screening occurred prior to the selection of participants, who were included if they: (1) were between 12–16 years old; (2) owned a personal mobile phone and (3) had a parent able to provide written informed consent.

### 2.2. Procedures

A fact sheet inviting participation was sent to parents three months prior to the intervention. Two weeks prior to the start of the intervention, all those who had provided written consent attended a briefing session and complete a baseline questionnaires which consisted of their socio-demographics, stage of change, perceived benefits and barriers of PA, and PA level. With this information, an algorithm of stage of change was used to identify their stage and messages matched with their respective stage of behavior were sent to them from the message bank which was developed in a pilot study [41]. Then, the tailored messages and phone numbers of each participant of the intervention group were entered to a commercial computerized texting system to set up a delivery schedule. The sender was set in the system by using their respective PE teachers’ name to provide a more solid support and motivation to the participants. All measures were assessed at baseline and 4-week (post intervention). The total duration of the intervention was four weeks.

**Figure 1:**
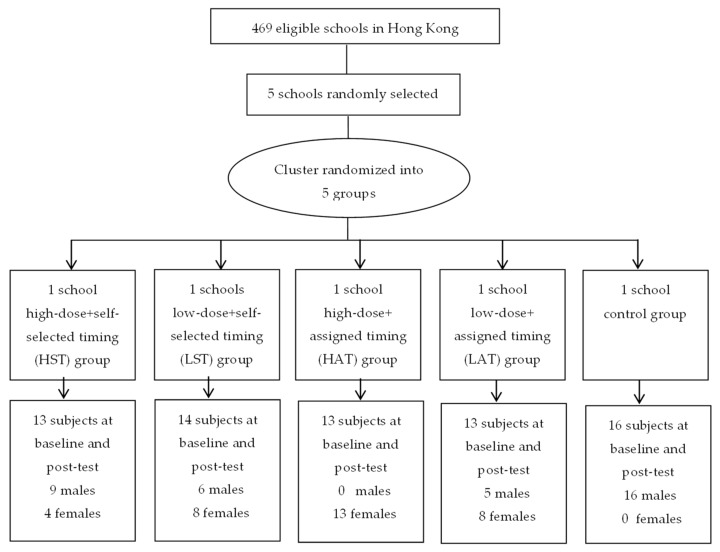
A CONSORT statement of participants’ flow.

This study used a randomized controlled cluster design. Invitations to participate in the study were sent to all government-subsidized secondary schools in Hong Kong. Among the 469 schools invitations, 10 schools replied and five schools were selected based upon the district categorizations (Hong Kong Island, Kowloon and New Territories) in Hong Kong. Each school was allocated through a ballot in which the five group labels (HST, LST, HAT, LAT, C) were picked. Participants were school students randomly assigned into one of the five groups (Figure 1):

(1) High-frequency + self-selected timing (HST): Participants received five SMS per week (on weekdays). Self-determined timing for the receipt of SMS (before school/afterschool/after dinner).
(2) Low-frequency + self-selected timing (LST): Participants received three SMS per week. Self-determined timing for the receipt of SMS.
(3) High-frequency + assigned timing (HAT): Participants received five SMS per week. Assigned timing for the receipt of SMS (after school).
(4) Low-frequency + assigned timing (LAT). Participants received three SMS per week. Assigned timing for the receipt of SMS (after school).
(5) Control group (C). No treatment.

### 2.3. Measures

Measures included: demographic information, self-reported PA, stage of behavior change and perceived benefits and barriers to PA. PA was measured with the Physical Activity Questionnaire for Children (PAQ-C) [42]. The PAQ-C is a self-administered, 7-day recall questionnaire for children aged 8 to 14 years composed of ten items, nine of which are structured to discern moderate-to-vigorous PA (MVPA) using a 5-point Likert scale with higher scores indicating higher PA levels. The summarized PAQ-C score is the average of the nine items scores. The PAQ-C score was the total score calculated by summing all nine items to indicate participant’s overall PA levels. The validation of Chinese version of the PAQ-C has been documented [43].

The stage of behavior change was measured using the Stage of Motivational Readiness Questionnaire [44], which was validated with the Seven-Day Physical Activity Recall [45]. This questionnaire contains four yes/no option items to determine participants’ readiness for PA and categorize them into different stages: pre-contemplation, contemplation, preparation, action, and maintenance. The questionnaire was translated into Chinese using the translation-back translation method and a pilot study was conducted. The Kappa index of reliability of 0.78 over a 2-week period was reported. To compare the movement status of groups related to the stage of motivational readiness, participants were divided into three categories (decreasing, stable and increasing). These category scores were determined by subtracting the pre-test from post-test scores.

Perceived benefits and barriers to PA were measured using the adolescent version of the Exercise benefits and barriers scale (EBBS) [44]. The term “exercise” in the original questionnaire is replaced by “PA” in order to reflect the PA context of the adolescents for the present study. The revised questionnaire was conducted to the participants and they showed no troubles to understand and respond to the questions related to their benefits and barriers to PA. This scale contained nine benefit and ten barrier items using a 5-point scale. The scale could also be separately transited into the benefits subscale and barriers subscale. The final score were the PA benefits and barriers scores, which were summed directly based on the benefits subscale and the barriers subscale and benefits/barriers differential score, which were calculated by subtracting the mean barriers score from the mean benefits score. Higher scores indicated greater perceived benefits or barriers or a differential score. A Cronbach’s alpha of 0.84 was reported for both benefits and barriers subscale. The Kappa indexes of the two subscales were 0.72 and 0.76, respectively.

### 2.4. Intervention

The tailored SMS messages were developed in a published pilot study [41]. According to the pilot study, four types of messages were created: (1) greeting (e.g., Hi I’m Jackie, what’s your name? Do you do PA regularly?), (2) motivation (e.g., Congratulations! You met your target last week. Great! I need to learn from you!), (3) exercise tips (e.g., Listen to the music and dance. It’s easy and nice for you.), and (4) reminder (e.g., shopping in and walking in a mall is also an exercise, please do it).

The content, language and tone of the SMS motivators were all tailored and matched according to the participant’s Stage of Motivational Readiness (SMR), PA benefit and PA barriers, as measured at the baseline assessment. The messages were written in colloquial language and a stage-appropriate tone. For the pre-contemplator, enabling them to understand the pros and cons of PA is the goal of this stage. The sample statement was “I can release stress and improve my health by doing simple PA like walking.” For the contemplators, the goal was to increase their likelihood to take action. A statement such as “Let’s try……” was used to stimulate the contemplators to think more and encourage them to do PA. The sample statement for those who want to improve his/her outlook (benefit), was “Let’s try to do some PA and improve my body posture and outlook”. Finally, for the participants in the preparation, action and maintenance stage, the goal was to increase their PA level to the recommended level. A directive statement was used to help these participants develop a PA plan. Therefore, “You can” was used in the sample statement for those who considered the distance of the PA venue excessively far (barrier), “If the PA venue is too far, you can stay home and do some sit-ups, rope skipping, and/or stretching. It’s better than doing nothing” (The President’s council on Physical Fitness and Sports, PCPFS, 2003) [44].

In addition, participants were told that messages would be sent by PE teachers. This was done to enhance social support because PE teachers are a known motivational influence affecting adolescent’s leisure time PA [46]. A commercial database (PCCW Mobile HK Limited, 2007) was used to record the usage of the SMS (number of SMS sent by the researcher and replied to by the participants).

### 2.5. Statistical Analysis

Baseline characteristics of the four intervention groups and the control group were compared using chi-square tests for categorical variables and the analysis of variance (ANOVA) for continuous variables. Analysis of covariance (ANCOVA) was conducted to test the intervention effect on the outcome controlling for age, gender, BMI and the baseline assessment of variable. To compare stage movement among groups, participants were divided into three categories (stable, progression to a higher stage, and regression to a lower stage), and these data were analyzed with chi-square tests. The level of significance was defined as *p* < 0.05; all tests were 2-tailed. All analyses were conducted using SPSS (version 16.0, SPSS Inc., Chicago, IL USA).

## 3. Results

### 3.1. Participants’ Characteristics

Participants’ baseline characteristics stratified by groups are presented in Table 1. Due to the small sample size, there were significant differences in gender (*χ*^2^ = 31.83, *p* < 0.001) and age (*χ*^2^ = 14.32, *p* = 0.006) distribution among groups. No significant differences were found in BMI among the five groups.

### 3.2. Intervention Effect on PA

The PAQ-C score at baseline and post-test in five groups are shown in Table 2. No significant intervention effects were detected among the five groups (F = 1.21, *p* = 0.421). However, an increasing trend was found in the HST and LAT groups. The PAQ-C score increased from 16.46 (SD: 6.54) at baseline to 18.75 (SD: 10.57) at post-test in the HST group as well as from 19.69 (SD: 8.59) at baseline to 21.85 (SD: 8.68) in the LAT group. However, this score decreased in the other four groups.

After intervention, five (38.5%) students in the HST group progressed to a higher stage, seven (53.8%) children remained at the same stage, and one (7.7%) child regressed to a lower stage. In the control group, the number of children that regressed to a lower stage (five children, 31.3%) was higher than those that progressed to a higher stage (three children, 18.8%) (Table 2). However, no significant differences were observed in the stage movement among the five groups (*χ*^2^ = 6.18, *p* = 0.627).

### 3.3. Intervention Effect on Exercise Benefits and Barriers

The exercise benefits, barriers and benefits/barriers differential scores are shown in Table 3. There were no significant differences among all the comparable groups (*p* > 0.05). However, we found a growth trend in the exercise benefits score in the LST and HAT groups compared with a downswing in the control group. The exercise barriers score in the HST group showed the largest reduction after intervention. The benefits/barriers differential score in all the intervention groups increased, whereas it decreased in the control group.

## 4. Discussion

### 4.1. What Is the Effect of SMS Delivery Frequency and Timing on Chinese Adolescents’ PA?

When the SMS frequency and timing issues are treated separately, it is reasonable to assume that the SMS frequency has a positive relation with adolescents’ PA (Table 4). Orr and colleagues [9] stated that a higher frequency of SMS (multiple messages a day) had a better effect on the health behavior change than a lower frequency. Rosenbaum et al. [47] suggested that the frequency of feedback could be a vital key to learn and/or to change perceptual-motor skills and intellectual skills. In the specific area of SMS dosage, optimal frequency information on SMS delivery in motivational and learning contexts has been lacking. However, this study did not demonstrate that the HST group could significantly increase adolescents’ PA levels, even the results showed the possible trend and meaningful impact of higher frequency and the self-selected timing of SMS on behavioral change. Due to the small sample size in this study, a future study should further explore the higher frequency and self-selected timing in the intervention design. This finding may offer initial support that five weekly, tailored SMS had a greater impact on self-reported PA than three weekly SMS and the control group. This change trend is consistent with the systematic review [40] on behavior change interventions delivered by SMS. The researchers found that the frequency of SMS transmission reflected the frequency of the target behavior for all except for two (twelve of fourteen) studies in the review. Newton et al. [29] also indicated that the low frequency of SMS (one message per week) could be the reason that PA behavior change intervention (12-week SMS and pedometer monitoring) did not produce any significant increase in step counts.

When considering the timing with the frequency, the self-selected (HST) and assigned timing (HAT) groups did not demonstrate any statistically significant change in PA levels (as represented by the PAQ-C score). However, an improvement trend was shown in the self-chosen time group when compared with the assigned time group and the control group. Crutzen, Cyr & de Vries [46] stated that, if participants had more control over their internet-based intervention, this may provide a positive effect to change their health behaviors. Tailored SMS are considered to be more effective in PA behavior change than untailored or generic bulk messages [40]. Autonomy of SMS timing could be part of the tailored SMS in which it could fit well into the modern daily life pattern or pace in different cities.

### 4.2. Were the Stages of Motivational Readiness Affected As a Result of the SMS Intervention?

No significant change occurred in the stage of motivational readiness over the course of the intervention among the five groups. De Bourdeaudhuij and colleagues [35] suggested that stages of PA behavior change are related to the frequency of encouragement from participants’ family and friends. The researchers revealed that the adolescents in the pre-contemplation stage reported the least social support from family and friends. This finding indicated that more social support and encouragement is needed to advance these physically inactive adolescents to the contemplation, preparation and action stages. In this case, SMS could be a desirable option to reach adolescents using a more convenient and cost-saving approach.

Regarding the SMS delivery timing, this finding may imply that the participants did not observe the relevance between the stage of behavior change and the SMS delivery time. Alternatively, certain pre-requisites might need to be met before the timing could be meaningful to the participants. The finding could also relate to the different stages the participants were at. Because no previous research attempted to investigate the impact of the timing of SMS delivery on adolescents’ PA behavior, this question could be further examined in a future study.

### 4.3. Was There a Difference in Perceived Benefits and Barriers As a Result of the SMS Intervention?

Despite insignificant results, the benefits/barriers differential score in all the intervention groups increased, although it decreased in the control group. This finding may suggest a potential value of SMS to the adolescents’ PA when considering the exercise benefits and barriers. Additionally, much larger differences were found in HST for perceived barriers when compared with the small decrease in the control group. More specifically, high frequency and self-selected timing had the largest impact on exercise barriers.

Grubbs and Carter [48] found a significant association between exercise benefits and barriers to individual’s exercise behavior and suggested that a deeper understanding of exercise benefits and barriers would contribute to the exercise behavior establishment. In the study by Blanchard et al. [49], the researchers suggested that perceived barriers’ efficacy may produce a more significant impact to explain PA behavior compared with task efficacy. Therefore, the possible effect of high frequency and self-selected timing of SMS on exercise barriers deserves further study to examine whether it could produce significant behavior change in a longer term intervention. O’Dea [50] suggested providing more social support, education and information on environment restructuring, planning, time management, motivation and varieties of PA for the school children to overcome the exercise barriers. High frequency and self-selected timing of SMS may be the instruments to help achieve this objective through mHealth technology.

## 5. Conclusions

To conclude, no significant intervention effects were found among the five groups in PA behavior, stage of change and exercise benefits and barriers among Hong Kong Chinese adolescents.

According to a systematic review on the health behavioral change and mode of delivery by e-intervention, Webb and his colleagues [51] suggested that greater use of supplementary modes, such as SMS, email, telephone, CD-ROM, or videoconferencing, could produce a better intervention effect. Another review was conducted to investigate the intervention effect of SMS in health behavioral change studies [52]. The review demonstrated that 29 of 34 SMS studies (85%) found that SMS played a more significant role and produced a positive effect on the participants when combined with internet and other strategies (e.g., telephone and computer program). The results suggested that the combined usage of SMS with other behavioral change strategies would produce more significant and positive effects for future interventions. In this case, more combined usage of SMS and other e-intervention modes of delivery, including computer programs, internet website, and emails, may produce a better effect on PA behavioral change.

The findings of this study should be interpreted with caution. First, this study used the self-reported data (PAQ-C) from the adolescents, and these data could be overestimated based upon the self-reported PA literature. Future studies should use the accelerometer to collect the objective PA data of the adolescents; thus, the analysis and findings could be more accurate. Second, it was a small-scaled study (*n* = 69); therefore, the generalizability of the findings is limited. Third, the relative short (4 week) intervention duration of this study may not allow for full conclusions to be drawn regarding the effects of the frequency and timing of SMS delivery on the adolescents’ PA behavioral change. Therefore, this issue should be addressed in the future. With the changes of PAQ-C in the HST and control groups after and before the intervention, power analysis result showed that the current sample size (*n* = 13 in the HST group and *n* = 16 in the control group) only provided the power of 0.71 at *p* set as 0.05. Due to the small sample size, this study was underpowered to detect a significant treatment effect. Further research should be conducted in a large representative sample in more schools and covering all grades to confirm our findings. Finally, future research may consider qualitative investigations to elaborate on the relations between the stages of change, perceived benefits and barriers, and the different frequency and timing of SMS delivery.

## Figures and Tables

**Table 1:** Participants’ characteristics.

Groups	*n*	Gender *n* (%)		Age (years) *n* (%)		Body Mass Index (kg/m^2^) Mean (SD)
		Male	Female	12–13	14–16	
HST	13	9 (69.2)	4 (30.8)	8 (61.5)	5 (38.5)	20.32 (4.30)
LST	14	6 (42.9)	8 (57.1)	3 (21.4)	11 (78.6)	20.07 (2.60)
HAT	13	0 (0.0)	13 (100.0)	9 (69.2)	4 (30.8)	20.95 (2.95)
LAT	13	5 (38.5)	8 (61.5)	6 (46.2)	7 (53.8)	20.92 (3.59)
Control	16	16 (100.0)	0 (0.0)	2 (12.5)	14 (87.5)	20.51 (3.56)

Notes: HST, High-frequency + self-selected timing; LST, Low-frequency + self-selected timing; HAT, High-frequency + assigned timing; and LAT, Low-frequency + assigned timing.

**Table 2:** Participants’ PAQ-C score and stage movement stratified by group and time.

Group	*n*	PAQ-C Total Score (Mean (SD))		*n*	Stage Movement (*n* (%))		
		Baseline	Post Test		Regression to a Lower Stage	Stable	Progression to a Higher Stage
HST	13	16.46 (6.54)	18.75 (10.57)	13	1 (7.7)	7 (53.8)	5 (38.5)
LST	14	21.31 (11.58)	18.31 (12.99)	14	3 (23.1)	4 (30.8)	6 (46.2)
HAT	13	22.46 (12.84)	20.38 (12.22)	13	3 (23.1)	5 (38.5)	5 (38.5)
LAT	13	19.69 (8.59)	21.85 (8.68)	13	2 (15.4)	8 (61.5)	3 (23.1)
Control	13	27.18 (13.40)	23.81 (13.34)	16	5 (31.3)	8 (50.0)	3 (18.8)

Notes: HST, High-frequency + self-selected timing; LST, Low-frequency + self-selected timing; HAT, High-frequency + assigned timing; LAT, Low-frequency + assigned timing; and PAQ-C, Physical activity questionnaire for children.

**Table 3:** Mean (SD) for measures stratified by group and time.

Group	*n*	Exercise Benefit Score		Exercise Barrier Score		Benefits/Barriers Differential Score	
		Baseline	Post Test	Baseline	Post-Test	Baseline	Post-Test
HST	13	27.54 (6.30)	27.08 (4.82)	28.80 (6.47)	24.33 (7.90)	−1.23 (7.81)	3.08 (8.48)
LST	14	33.38 (5.92)	36.69 (4.59)	29.93 (6.62)	30.62 (6.21)	3.08 (8.64)	5.25 (7.82)
HAT	13	34.00 (5.35)	35.00 (6.72)	30.69 (8.37)	27.15 (7.07)	3.31 (8.13)	7.85 (8.36)
LAT	13	31.62 (4.81)	30.42 (2.47)	28.08 (5.17)	26.08 (4.84)	3.54 (6.35)	3.67 (4.19)
Control	16	35.69 (5.02)	33.06 (5.77)	25.94 (7.08)	25.36 (8.56)	9.75 (10.61)	7.50 (12.47)

Notes: Data are presented as the mean (SD). HST, High-frequency + self-selected timing; LST, Low-frequency + self-selected timing; HAT, High-frequency + assigned timing; and LAT, Low-frequency + assigned timing.

**Table 4:** SMS Frequency and PAQ-C score.

Group	PAQ-C Total Score					
	*n*	Pre-Test		Post-Test		*p* Value *
		Mean	SD	Mean	SD	
Intensity						
High group	19	19.42	10.97	20.68	11.54	0.043
Low group	21	18.76	9.12	19.14	11.39	
Control group	13	27.18	13.40	23.81	13.34	

Notes: * Denotes *p* value analyzed using the Kruskal-Wallis h test for the post-test/pre-test differential score (calculated by subtracting the pre-test from the post-test score).
